# Microbial copper resistance: importance in biohydrometallurgy

**DOI:** 10.1111/1751-7915.12450

**Published:** 2016-10-28

**Authors:** Cristóbal Martínez‐Bussenius, Claudio A. Navarro, Carlos A. Jerez

**Affiliations:** ^1^ Laboratory of Molecular Microbiology and BiotechnologyDepartment of BiologyFaculty of SciencesUniversity of ChileSantiagoChile

## Abstract

Industrial biomining has been extensively used for many years to recover valuable metals such as copper, gold, uranium and others. Furthermore, microorganisms involved in these processes can also be used to bioremediate places contaminated with acid and metals. These uses are possible due to the great metal resistance that these extreme acidophilic microorganisms possess. In this review, the most recent findings related to copper resistance mechanisms of bacteria and archaea related to biohydrometallurgy are described. The recent search for novel metal resistance determinants is not only of scientific interest but also of industrial importance, as reflected by the genomic sequencing of microorganisms present in mining operations and the search of those bacteria with extreme metal resistance to improve the extraction processes used by the biomining companies.

## Introduction

Biomining is used to enhance the recovery of metals from ores such as sulfide minerals. Bioleaching solubilizes the metal of interest by biological catalysis as in the case of copper. On the other hand, during biooxidation microorganisms dissolve the mineral matrix occluding the metal to be extracted as it occurs in the case of gold (Kaksonen *et al*., 2014; Urbieta *et al*., 2015; Harrison, 2016). These mineral–microbe interactions to recover metals from ores have been widely used in small and large scales (Brierley and Brierley, 2013; Norris *et al*., 2013; Harrison, 2016). An acidophilic microbial consortium is in charge of metals mobilization. In practice, commercial biomining procedures are mainly used for copper bioleaching and the biooxidation of refractory gold ores (Brierley and Brierley, 2013; Kaksonen *et al*., 2014; Urbieta *et al*., 2015). During minerals bioleaching acid mine drainage is generated which can cause serious environmental problems. However, bioremediation or removal of the toxic metals from contaminated soils can be achieved by using abiotic methods or the specific properties of the acidophilic microorganisms interacting with these elements (Johnson and Hallberg, 2005).

Industrial biomining operations are of several kinds depending on the ore type and its geographical location, the metal content and the specific minerals present (metal oxides and mostly secondary metal sulfides such as covellite, chalcocite and others). Metal recovery processes can be carried out by using tank, heap, dump and *in situ* leaching systems (reviewed in detail by Harrison, 2016). One of the most used setups for the recovery of copper is the irrigation type of processes. These involve the percolation of leaching solutions through the crushed ore that can be contained in a column, heap or dump (Watling *et al*., 2010; Jerez, 2011; Brierley and Brierley, 2013). The pregnant solution containing copper sulfate generated by the microbial solubilization of the insoluble copper sulfides present in the ore, is subjected to solvent extraction to obtain a highly concentrated copper sulfate solution from which the metal is recovered in an electrowinning plant to generate electrolytic copper of high purity (Watling *et al*., 2010; Harrison, 2016).

On the other hand, as reactors are expensive to build, they are used with high‐grade ores or with mineral concentrates. The advantages of tank reactors over heaps and dumps, which are ‘open bioreactors’ is that in the tanks conditions can be controlled. The use of hyperthermophilic archaea for mineral sulfide processing has been studied mainly because they appear to have greater tolerances to high copper concentrations and solid particle concentrations that take place in bioreactors compared with other thermoacidophiles (Brierley and Brierley, 2013; Norris *et al*., 2013; Harrison, 2016). Therefore, the search for key efficient mineral‐oxidizing strains with very high metal resistance may be of great importance to develop improved industrial bioreactors for refractory minerals (Norris *et al*., 2013; Wheaton *et al*., 2015; Urbieta *et al*., 2015).

Thermophilic archaea are able to oxidize mineral sulfides at temperatures higher than 60°C. Among these, *Acidianus brierleyi*,* Sulfolobus metallicus* and *Metallosphaera* have been the most studied (Brierley and Murr, 1973; Lindström and Gunneriusson, 1990; Jones *et al*., 2011; Norris *et al*., 2013; Wheaton *et al*., 2015). When a microbial community is used to start a series of tanks for biomining, metal concentrations in the leachate increase and those species resistant to extremely high metal concentrations (as well as other compounds such as organic carbon) are selected in the final tank (Okibe *et al*., 2003; Dopson and Holmes, 2014). This suggests that those microbial species with higher metal resistances may be advantageous for the biomining process. In this regard, a spontaneous mutant strain of *Metallosphaera sedula* (CuR1) with supranormal metal resistance has been reported to leach copper from chalcopyrite (CuFeS_2_) at an accelerated rate (Maezato *et al*., 2012) (see the archaeal section below).

Several recent reviews related to biomining methods and the microorganisms involved are available (Brierley and Brierley, 2013; Norris *et al*., 2013; Vera *et al*., 2013; Kaksonen *et al*., 2014; Wheaton *et al*., 2015; Zhuang *et al*., 2015; Cárdenas *et al*., 2016; Harrison, 2016; Hedrich and Schippers, 2016). They cover bacteria and archaea involved in biohydrometallurgy, and describe the new findings obtained by using Omics techniques, such as genomic DNA sequencing of single species or metagenomic sequencing of the microbial communities involved, proteomics and metaproteomics and metabolomics (Martínez *et al*., 2015). The recently published book ‘Acidophiles: Life in Extremely Acidic Environments’ (Quatrini and Johnson, 2016) is also a reference of great interest as most of its chapters are related to aspects important for bioleaching.

A mini‐review related to several metals resistance in acidophilic microorganisms has been published before (Dopson and Holmes, 2014). In the present review, we concentrate in critically analysing the most recent findings related to copper resistance and its importance in copper sulfides bioleaching.

## Microorganisms and bioleaching communities

The most studied leaching bacteria are from the *Acidithiobacillia* class. *Acidithiobacillus ferrooxidans* and *A. thiooxidans* are acidophilic mesophiles and together with the moderate thermophile, *A. caldus*, are some of the most studied members of the biomining consortium. The role in biomining of many other acidophilic microorganisms present in natural and man‐made acidic environments has recently been reviewed (Hedrich and Schippers, 2016). Although *A. ferrooxidans* has been described to oxidize metal sulfides even at 0°C (Langdahl and Ingvorsen, 1997), no psychrophilic acidophiles (optimal growth temperature lower than 15°C) have been reported. Some psychrotolerant microorganisms such as *Acidithiobacillus ferrivorans* have been also isolated that can catalyse metal dissolution from sulfide minerals at 5°C. These bacteria are of importance in biomining operations as several of them are present at high altitudes and in Polar regions and therefore can be used for biotechnological operations in cold environments (Dopson, 2016).

Chalcopyrite is the most abundant copper‐containing mineral (around 70% of the world's copper reserves). However, it is the most difficult mineral to solubilize by mesophilic microorganisms, taking much longer time to recover copper. Therefore, there is actually great interest in developing processes by using thermophilic biomining microorganisms for these recalcitrant minerals.

## Genetic manipulation and recent advances using OMICS to analyse biomining microorganisms

It has been postulated that it is important to know the copper resistance mechanisms of bioleaching microorganisms in order to construct strains with high copper tolerance by using genetic modification (Wen *et al*., 2014). Transformation of some biomining microorganisms has been possible. Gene knockout systems were previously reported in the case of *A. ferrooxidans* and *A. caldus* (Liu *et al*., 2000; Van Zyl *et al*., 2008). These transformations were performed by using a method based on marker exchange mutagenesis. Later on, an improved method of markerless gene replacement was established for *A. ferrooxidans* (Wang *et al*., 2012). However, the reported procedures are difficult to reproduce, very laborious and have very low efficiencies. Recently, a versatile and efficient markerless gene knockout system for *A. thiooxidans* was reported and used to characterize a copper tolerance related multicopper oxidase (MCO) gene in this microorganism (Wen *et al*., 2014).

Instead, some authors consider that the engineering constraints of all forms of biomining operations (including stirred tanks) means that it is not possible to prevent the release of microorganisms used in these processes to the environment (Johnson, 2014). An alternative to this situation is the selection of microorganisms from biomining areas that possess a better natural copper resistance together with a higher capacity to bioleach mineral sulfides. *A. ferrooxidans* strain ATCC 53993 might be an example of this kind of bacteria, as it is able to resist high copper concentrations (10–12 g L^−1^ range) (Orellana and Jerez, 2011) and capable of oxidizing both reduced inorganic sulfur compounds (RISCs) and ferrous iron. In the case of *A. thiooxidans* and *A. caldus*, as they oxidize only RISCs, an additional strain such as *Leptospirillum ferrooxidans* or a similar ferrous iron oxidizer would be required for suitable bioleaching of metal sulfides. Some biomining companies such as BioSigma, S.A. are currently using this approach and have isolated native species from copper mines such as *A. ferrooxidans* strain Wenelen (DSM 16786) and others with increased activity for copper leaching from mixed sulfide ores and high copper resistance (higher than 10 g L^−1^ compared with 5 g L^−1^ of the type *A. ferrooxidans* ATCC 23270 strain) for their use in industrial processes (Sugio *et al*., 2009; Latorre *et al*., 2016).

Proteomics, metaproteomics, genomics and metagenomics, together with metabolomics, have been widely used in recent years to study the global regulatory responses including metal resistance systems of all kinds of cells individually or in complex microbial communities (Gillan, 2016). By this approach it may be possible to explore the new properties of biomining microorganisms that arise from the interplay of genes, proteins, other macromolecules, small molecules and the environment. These procedures enable to build metabolic models, predict microbial interactions, defining genetic diversity and study microbial evolution. Some of the findings obtained by using these molecular procedures for the study of biomining microorganisms and their metal resistance have been recently reviewed (Martínez *et al*., 2015; Cárdenas *et al*., 2016). Genomics has been of great importance to define the biodiversity of biomining processes and has allowed developing both conceptual and metabolic models for some of the acidophilic microorganisms involved. By using metabolomics, some specific metabolites may be used as markers to follow the evolution in time of industrial bioleaching operations (Martínez *et al*., 2013). On the other hand, proteomics has been useful to study microorganisms–mineral interactions and biofilm formation (Martínez *et al*., 2015), including copper resistance as described in the next section.

Global changes in gene expression and metabolite levels allow exploring both cannonical and new possible mechanisms of metal resistance and also likely unknown passive or active metal tolerance systems. It is expected that a detailed knowledge of the responses that these environmental microorganisms use to adapt to their harsh niche will help to improve biomining and metal bioremediation in industrial processes (Jerez, 2013; Martínez *et al*., 2015).

## Copper resistance mechanisms

Acid‐leaching solutions are characterized by concentrations of base and transition metals that are toxic to most living organisms, and as might be expected, microorganisms that grow in mineral‐rich environments are, in most cases, remarkably tolerant to a wide range of metal ions and should possess robust metal resistance mechanisms (Orell *et al*., 2012; Dopson and Holmes, 2014).

Biomining bacteria and archaea resist high levels of copper (100–300 mM range) by using a few ‘canonical systems’ such as active efflux or trapping of the metal ions by metal chaperones. Nonetheless, gene duplications, the presence of genomic islands (GI), the existence of additional strategies such as passive mechanisms for pH and cations homeostasis in acidophiles, and an inorganic polyphosphate (polyP)‐driven metal resistance mechanism have also been proposed (Chi *et al*., 2007; Orell *et al*., 2010, 2012; Orellana and Jerez, 2011; Navarro *et al*., 2013; Dopson and Holmes, 2014; Wen *et al*., 2014; Wheaton *et al*., 2015). Next a selection of the most studied biomining microorganisms is analysed regarding their copper resistance capacity.

## Copper resistance of biomining bacteria

### Acidithiobacillus ferrooxidans

*Acidithiobacillus ferrooxidans* ATCC 23270 can survive under high copper concentrations. It does so by carrying in its genome more than 10 genes related to copper homeostasis in other bacteria (Rensing and Grass, 2003; Nikaido, 2011). Three of these genes code for putative ATPases related to the transport of copper (*copA1*_*Af*_, *copA2*_*Af*_ and *copB*_*Af*_), three other genes are related to a system of the RND family, involved in the extraction of copper from the cell by using the proton motive force (PMF; *cusA*_*Af*_, *cusB*_*Af*_, *cusC*_*Af*_) and two additional genes code for periplasmic chaperones for this metal (*cusF*_*Af*_ and *copC*_*Af*_) (Navarro *et al*., 2009). These *A. ferrooxidans* copper resistance determinants were found to be upregulated when this bacterium was exposed to CuSO_4_ in the range of 5–25 mM and conferred a greater resistance to copper when expressed in *Escherichia coli* compared with wild‐type cells, supporting their functionality (Navarro *et al*., 2009).

*Acidithiobacillus ferrooxidans* and other acidophiles live at an acid external pH (1–3) and their cytoplasmic pH is up to 5 units higher than the external pH. This generates an elevated pH gradient across the cytoplasmic membrane that contributes to the PMF comprising the membrane potential (ΔΨ) and the transmembrane pH difference (ΔpH) (Baker‐Austin and Dopson, 2007). The RND‐type transporters are antiporters taking advantage of the protons gradient for the efflux of copper with protons entrance to the cytoplasm. Due to its economy from the energetic point of view, the acidophilic microorganisms would preferentially use these systems to remove intracellular copper. A possible cytoplasmic acidification would be expected to take place if the microorganisms would excessively use these efflux pumps in the presence of high metal concentration. However, this acidification could be diminished by the energetic metabolism of the bacterium, as the oxidation of Fe(II) or reduced sulfur compounds by molecular oxygen as the final electron acceptor consumes protons. As the RND systems are introducing protons from the culture medium to the cell during copper detoxification, an increase in the extracellular pH of the growth medium would be expected in the presence of this metal.

The genomic sequence of *A. ferrooxidans* ATCC 53993 contains all the copper resistance genes present in *A. ferrooxidans* ATCC 23270 that have been experimentally confirmed as being expressed in the presence of copper, except that strain ATCC 23270 contains a second Cus‐like operon interrupted by a transposase gene which is absent in the genome of strain ATCC 53993 (Luo *et al*., 2008; Navarro *et al*., 2009; Orell *et al*., 2010; Orellana and Jerez, 2011). These ORFs have 100% identity between their corresponding DNA sequences. However, *A. ferrooxidans* ATCC 53993 contains several additional putative metal resistance ORFs that confer it a higher metal resistance (Orellana and Jerez, 2011). These putative genes are clustered in a 160 kb GI that is absent in the genome of *A. ferrooxidans* strain ATCC 23270 (Cárdenas *et al*., 2010; Orell *et al*., 2010). The GI in *A. ferrooxidans* ATCC 53993 contains genes encoding heavy metal resistance determinants for copper, mercury detoxification and volatilization, and the transcriptional regulator (*merR*), as well as a copper translocating P‐type ATPase. On the other hand, strain ATCC 23270 has a 300 kb region that does not contain known metal resistance‐related genes and is absent in the genome of strain ATCC 53993 (Cárdenas *et al*., 2010; Bustamante *et al*., 2012). Obviously, the presence of unknown metal resistance genes in this 300 kb GI cannot be excluded at present.

Six ORFs possibly related to Cu resistance are present in the GI of strain ATCC 53993, including a P‐type ATPase annotated as a possible Cu resistance determinant or *copA3*_*Af*_ (Orellana and Jerez, 2011). All Cop‐like proteins found so far in *A. ferrooxidans*, including CopA3_Af_ contain the metal‐binding motifs that are highly conserved in Cop proteins from other microorganisms (Orell *et al*., 2010).

**Figure 1:**
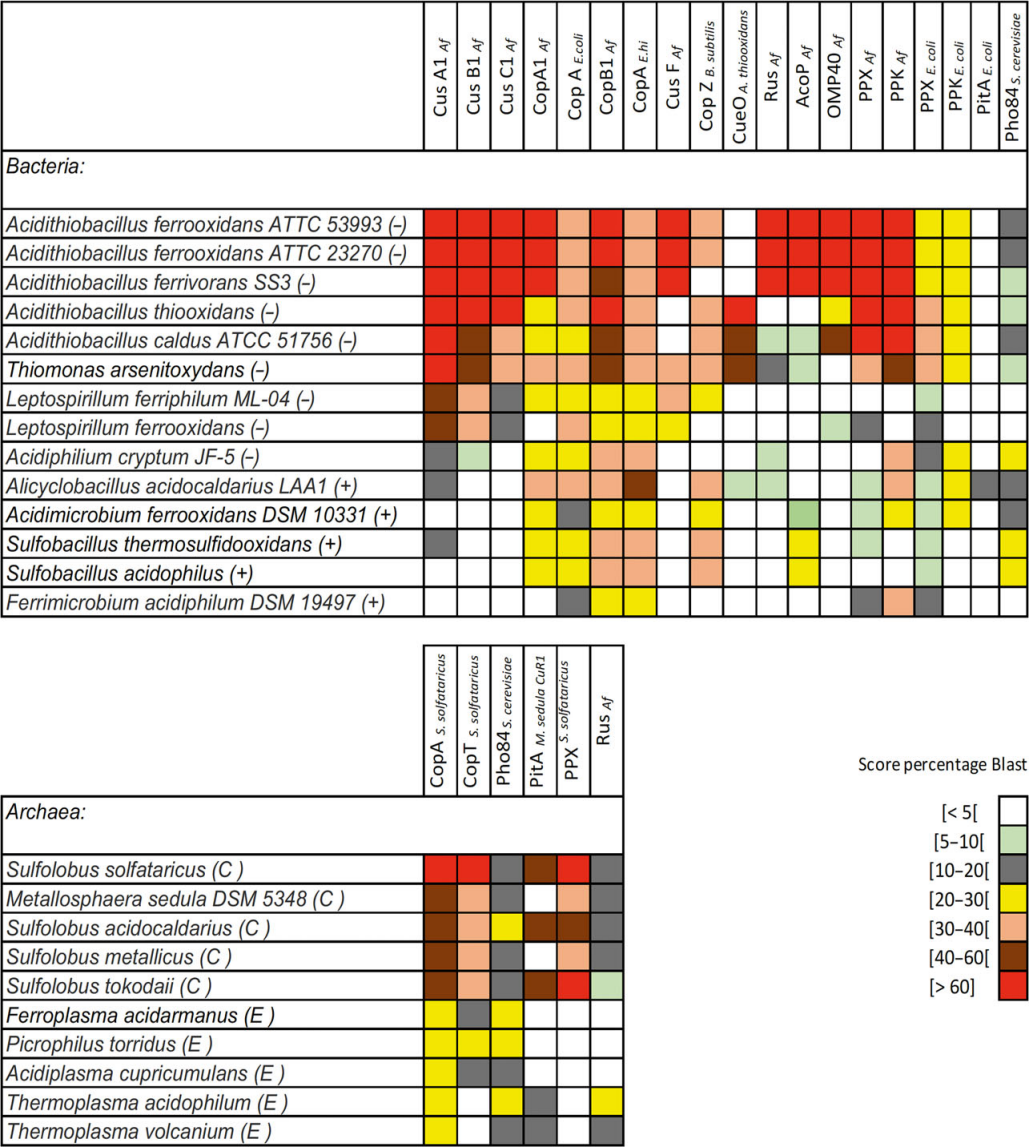
Comparison of the main copper resistance determinants present in a selection of acidophilic bacteria and archaea usually found in biomining environments. All the microorganisms chosen have their genomes sequenced and are publically available. The proteins selected for comparison are those for which at least some experimental evidence as copper resistance determinants exists. Symbols: (−) Gram negative; (+) Gram positive; (C) Crenarchaeota; (E) Euryarchaeota. The Blastp program was used to align protein sequences by means of BLOSUM62 substitution matrix. The colours scale indicate the percentage of the obtained scores related to the maximum score value obtained. The entrance protein sequences are in each column of the table and when more than one protein with a significant score was present in the same microorganism the highest score was taken in each case. Af, Acidithiobacillus *ferrooxidans*; E. hi, *Enteroccoccus hirae*.

Other of the ORFs found in the GI of *A. ferrooxidans* ATCC 53993 corresponded to members of a putative Cus system (*cusA3*_*Af*_, *cusB3*_*Af*_ and *cusC3*_*Af*_). Two additional possible CusF‐like chaperones previously described in *A. ferrooxidans* ATCC 23270 (Navarro *et al*., 2009) were found in this GI (Orellana and Jerez, 2011). A comparison of the possible copper resistance genes between these two *A. ferrooxidans* strains and several other selected biomining microorganisms is shown in Figure 1.

*Acidithiobacillus ferrooxidans* ATCC 53993 has a much higher resistance to CuSO_4_ (>100 mM) than that of strain ATCC 23270 (<40 mM) (Orellana and Jerez, 2011). As it occurs in *Cupriavidus metallidurans* (Van Houdt *et al*., 2009), it was expected that the additional multiplicity of possible copper resistance determinants in the GI of *A. ferrooxidans* ATCC 53993 would confer it a higher tolerance to the metal compared with strain ATCC 23270. Although both strains have a similar growth in the absence of copper, cell numbers of ATCC 23270 were reduced by sevenfold when grown in 100 mM CuSO_4_, whereas those of ATCC 53993 were diminished by only twofold (Orellana and Jerez, 2011; Jerez, 2013). This additional capacity of strain ATCC 53993 to tolerate copper should confer it an adaptational advantage when growing in a microbial consortium such as the one usually found in their habitat. If these two *A. ferrooxidans* strains were present in a bioleaching operation such as a heap or more likely in an industrial biooxidation reactor, as copper concentration increases with the dissolution of the mineral, at the end of the operation strain ATCC 53993 should predominate over ATCC 23270. A preliminary test for this idea was carried out by growing a mixture of approximately equivalent numbers of each strain in the presence of copper. The relative proportions of each kind of bacterium were estimated by means of qPCR by using strain‐specific primers. The results obtained clearly indicated that *A. ferrooxidans* ATCC 53993 was able to outgrow strain ATCC 23270 in the presence of 50 mM CuSO_4_ (Orellana and Jerez, 2011; Jerez, 2013).

Moreover, an upregulation of the transcriptional expression of most of the additional Cu resistance genes present in the GI of *A. ferrooxidans* ATCC 53993 was observed when cells were grown in the presence of increasing CuSO_4_ concentrations. In addition, these genes were functional when expressed in *E. coli*, strongly supporting the functionality of the copper resistance determinants present in the GI of *A. ferrooxidans* ATCC 53993 (Orellana and Jerez, 2011). These findings constituted the first experimental evidence for high copper resistance due to the expression of genes present in a GI of an acidophilic chemolithoautotrophic bacterium.

Interestingly, when *A. ferrooxidans* strains ATCC 53993 vs ATCC 23270 subjected to 40 mM copper were analysed by differential quantitative proteomics, only 49 proteins changed in the former strain vs 120 proteins in the latter one (Martínez‐Bussenius *et al*., 2016). This greatly diminished response to the metal is in good agreement with the much higher copper resistance of *A. ferrooxidans* ATCC 53993 already described. These inter‐population interactions may not only lead to changes in the capacities of the bacteria to adapt to their environment but may also help to select the more suitable microorganisms to enhance industrial biomining operations.

Genomic strain variations have also been found in the acidophilic *Leptospirillum* group II by deep metagenomic genome sequence analysis (Simmons *et al*., 2008). It is possible that horizontal gene transfer between *A. ferrooxidans* strains and other microorganisms are key elements to supplement metal resistance and possibly other properties, thus conferring adaptational advantages to all these microorganisms.

One of the most abundant proteins in ferrous iron‐grown *A. ferrooxidans* is the cupredoxin rusticyanin (Rus) as it occupies 21% of the total volume in the periplasmic space (Li *et al*., 2015). This periplasmic component forms part of an iron‐oxidizing/oxygen‐reducing supercomplex spanning both inner and outer membranes in *A. ferrooxidans* (Yarzával *et al*., 2004; Castelle *et al*., 2008). Rus and other components of the *rus* operon have also been found expressed at lower levels in sulfur‐grown cells (Ramírez *et al*., 2004; Yarzával *et al*., 2004). However, the role for Rus in sulfur‐grown cells is still not clearly defined (Yarzával *et al*., 2004). Thus, the overexpression of Rus in sulfur‐grown cells exposed to copper supports the idea that this copper‐binding protein and possibly other components of the *rus* operon may have an additional role in *A. ferrooxidans* copper resistance, as previously suggested (Felício *et al*., 2003), and recently supported by Almárcegui *et al*. (2014a). In this regard, the analysis of the *A. ferrooxidans* periplasm by high‐throughput proteomics including high‐resolution linear ion trap‐FT MS allowed the identification of 131 periplasmic proteins (Chi *et al*., 2007). Among these proteins, several were uncharacterized, including AFE_3151. This protein was later proposed to be part of the iron‐oxidizing/O_2_‐reducing system in *A. ferrooxidans* although its role has not been defined so far (Castelle *et al*., 2008, 2010). This periplasmic protein, now named AcoP, has recently been characterized and it also contains a cupredoxin type copper‐binding site that binds one Cu atom per molecule and likewise forms part of the *rus* operon and iron respiratory system (Roger *et al*., 2014). Furthermore, the levels of AcoP also increased in sulfur‐grown cells in the presence of copper (Almárcegui *et al*., 2014a). Rus and AcoP have conserved copper‐binding sites (HXCX_2‐4_HX_4_M and HXCX_4‐8_HX_4_M respectively) (Roger *et al*., 2014) such as those described in CopC, a cupredoxin‐like copper‐binding protein from *P. syringae* involved in copper homeostasis (Arnesano *et al*., 2002).

By using high‐throughput quantitative ICPL proteomics, it was found that *A. ferrooxidans* ATCC 23270 grown in ferrous iron or elemental sulfur and in the presence of 40 mM of copper sulfate changes the expression of several proteins related to copper response, such as the upregulation of a Cus system (Almárcegui *et al*., 2014a,b), confirming the previously reported transcriptional analysis (Navarro *et al*., 2009). Furthermore, the upregulation (almost 15‐fold) of a protein coded by AFE_1862, annotated in the genome of *A. ferrooxidans* ATCC 23270 as a putative heavy metal‐binding protein were also detected by the proteomic approach (Almárcegui *et al*., 2014b). This protein contains a possible heavy metal‐binding domain (MXCXXC) similar to the one found in CopZ‐like proteins.

The possible role of Rus and AcoP periplasmic cupredoxins and of the putative cytoplasmic CopZ in copper resistance in *A. ferrooxidans* was recently reported (Navarro *et al*., 2016). Although CopZ is a known cytoplasmic copper chaperone that binds and delivers copper to CopA in *B. subtilis* (Radford *et al*., 2003), the CopZ‐like protein of *A. ferrooxidans* ATCC 23270 is the first cytoplasmic copper chaperone described in a biomining bacterium with a role in copper resistance. Likewise, Rus and AcoP cupredoxins apparently function not only as components of the ferrous iron oxidation pathway in this acidophilic microorganism but they may also bind excess copper in the periplasm, most likely playing a role in the considerably high copper resistance of *A. ferrooxidans* (Navarro *et al*., 2016).

An increased time of growth has been observed for ferrous iron‐grown *A. ferrooxidans* cells in the presence of copper, suggesting that respiration is affected and the oxidation of ferrous iron occurs more slowly in the presence of the metal (Almárcegui *et al*., 2014b). It is well documented in the literature that ferrous iron oxidation decreases in several *A. ferrooxidans* strains when copper concentration increases (Leduc *et al*., 1997). The results recently reported by Navarro *et al*., 2016 showed that the two proteins AcoP and Rus are upregulated in *A. ferrooxidans* cells in the presence of copper, suggesting they might have an additional role in copper tolerance. Rus could be eventually saturated with the bound copper. However, the extremely high concentration of Rus is in the range of copper sulfate that could be present under the usual growth condition for this acidophile. Rus copper‐trapping would be only one of many other components that respond to the presence of the toxic metal in the cells.

It is known that in aerobic systems Cu(II) supplied is reduced to Cu(I) upon contact with the respiratory chain of bacteria (Thauer *et al*., 1977; Volentini *et al*., 2011). Almost certainly reduced quinones are the electron donors. As Cu(I) is much more toxic than Cu(II), it could be exported from the periplasm to the outside by Cus‐like RND systems or oxidized back to Cu(II) by CueO/PcoA/CopA‐like periplasmic copper‐containing copper oxidases. *A. ferrooxidans* has Cus‐like RND systems (Navarro *et al*., 2009) but it apparently lacks genes coding for periplasmic copper‐containing copper oxidases. A new interesting speculative possibility for this acidophile to prevent Cu(I) extreme toxicity would be to transfer an electron from Cu(I) to the iron oxidation system (Navarro *et al*., 2016). For this it would be necessary that ApoRus and/or apoAcoP are able to bind Cu(I) at the periplasm. In this connection, it is known that folded aporusticyanin rapidly binds Cu(I) with much higher affinity than Cu(II) (Alcaraz *et al*., 2007). As proposed by Li *et al*. (2015), the concentrated Rus in the periplasm would constitute a network where inter‐protein electron transfer interactions across the periplasmic space would effectively function in connection with the respirasome. As previously suggested, it is not unthinkable that this copper‐binding protein may also constitute in part a barrier to trap excess copper in a microorganism normally confronted with high concentrations of this metal (Navarro *et al*., 2016).

**Figure 2:**
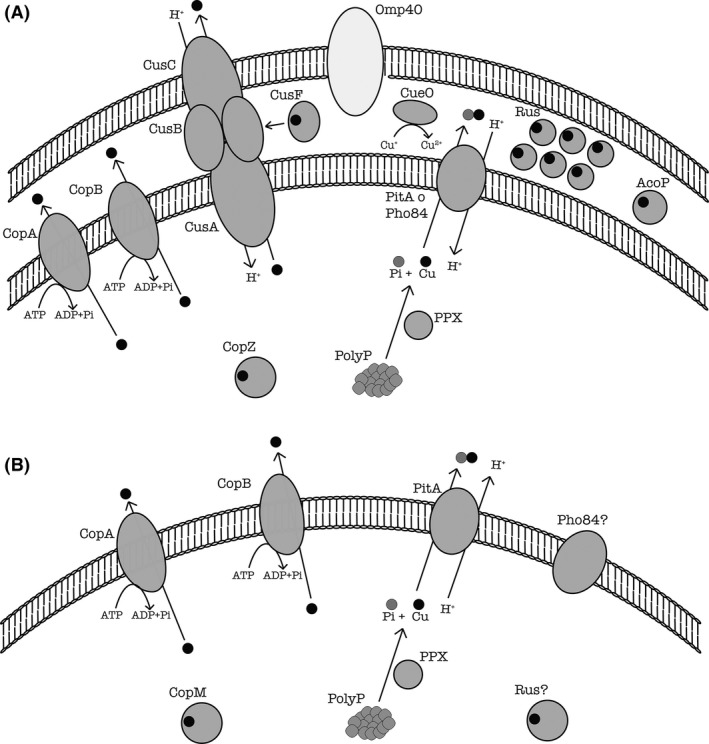
Summary working model of copper resistance determinants in biomining bacteria (A) and archaea (B). Dark grey, proteins that increase their synthesis levels in cells subjected to copper; light grey those down expressed in the presence of copper.

A working model including the possible role of Rus and possibly AcoP in copper resistance is shown in Fig. 2A. Obviously much more experimental work will be required to support these proposals. Not all biomining microorganisms contain Rus (Fig. 1), but in those containing Rus‐like proteins coding genes, such as *A. ferrivorans*, a similar role for this copper‐binding protein could be possible, especially considering that they possess the same copper‐binding site of type I found in *A. ferrooxidans* Rus. On the other hand, in sulfur‐oxidizing microorganisms such as *A. thiooxidans* and *A. caldus* that lack *rus* genes (Fig. 1) and are less resistant to copper compared with *A. ferrooxidans*, different components for copper resistance may be expected.

The copper‐responsive protein complement of iron‐grown *A. ferrooxidans* ATCC 23270 cells was recently evaluated by using ICPL proteomics (Almárcegui *et al*., 2014b). In addition of the expected upregulation of RND‐type Cus systems, some different RND‐type efflux pumps were overexpressed together with the upregulation of several genes involved in histidine and cysteine synthesis. In response to copper, a downregulation of the major outer membrane porin (Guiliani and Jerez, 2000) and some ionic transporters was observed in *A. ferrooxidans* ATCC 23270 cells grown in ferrous iron or sulfur (Almárcegui *et al*., 2014a,b). This result is an example of how a downregulation of outer membrane porins could cause a general decrease in the influx of the metal into the cell.

The native *A. ferrooxidans* strain Wenelen (DSM 16786) isolated from an industrial copper biomining operation in Chile by Biosigma S.A. was recently reported to have an extreme capacity to resist high concentration of copper (over 10 g L^−1^) compared with the type strain ATCC 23270 (5 g L^−1^). Furthermore, the global transcriptional response of this native strain showed the upregulation of several genes related with copper tolerance when the bacterium was grown in the presence of pyrite and CuFeS_2_ (Latorre *et al*., 2016).

Current knowledge indicates that in addition to the key elements involved in *A. ferrooxidans* copper resistance already discussed, an abundant reserve of inorganic polyP used as a polyP‐based copper resistance system and a robust defensive response to oxidative stress are also important (Alvarez and Jerez, 2004; Remonsellez *et al*., 2006; Orell *et al*., 2010). Poly P is synthesized in bacteria by a polyphosphate kinase (PPK) and degraded by a polyphosphatase (PPX) (Kornberg *et al*., 1999). The presence of copper in the cytoplasm would activate PPX, which would degrade polyP to generate free phosphate (see Fig. 2). This phosphate can bind copper to be eliminated to the periplasm or the extracellular medium by means of phosphate transporters such as PitA as proposed before for *E. coli* (Keasling, 1997; Van Dien *et al*., 1997) and recently demonstrated for *E. coli* by Grillo‐Puertas *et al*. (2014). Figure 2A summarizes a general working model for copper resistance in *A. ferrooxidans* and other acidophilic bacteria.

### Acidithiobacillus ferrivorans

**Figure 3:**
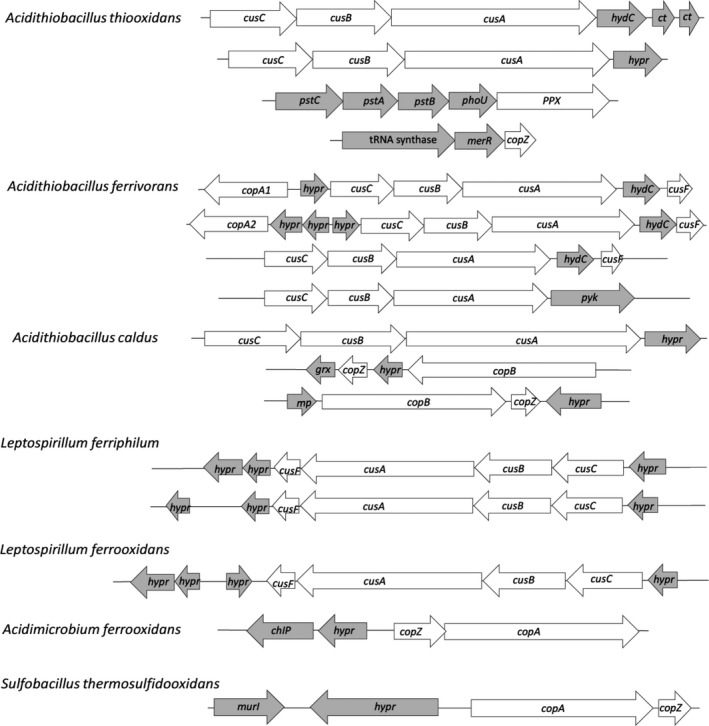
Gene clusters of some copper resistance determinants as predicted by bioinformatics analysis of the indicated selected microorganisms genome sequences. *ct*, cation transporter; *hypr*, hypothetical protein; *hydC*, cytochrome b561; *pyk*, pyruvate kinase C; *merR*, transcriptional activators; *pstS*, phosphate ABC transporter substrate‐binding protein; *pstA*, phosphate ABC transporter, permease protein; *pstB*, phosphate ABC transporter ATP‐binding protein; *phoU*, phosphate transport system regulatory; *grx*, glutaredoxins; mp, membrane protein; *chlP*, geranylgeranyl reductase; *murl*, glutamate racemase.

As previously described by González *et al*. (2014), *A. ferrivorans* SS3 genome contains four possible *cus* clusters, three of them *cusCBAF* type and a fourth one of *cusCBA* type. All *A. ferrivorans* SS3 putative Cus systems showed a high score of identity to the *A. ferrooxidans* system (over 75% Blastp score). Furthermore, upstream of the genomic context of two of the Cus systems of *A. ferrivorans* SS3, there is a putative copper ATPase (Fig. 3) with 95% Blastp score with CopA from *A. ferrooxidans* (Fig. 1).

The *A. ferrivorans* SS3 genome contains two additional copies of the *rus* gene (Liljeqvist *et al*., 2013) and its three Rus proteins not only have a high Blastp score (>85%) to Rus from *A. ferrooxidans* but all of them also conserve the same copper‐binding site. RusA from A. *ferrivorans* SS3 is the protein with the highest identity compared with Rus from *A. ferroxidans* and it forms part of the *rus* operon from *A. ferrivorans* together with the gene coding for the AcoP protein (Liljeqvist *et al*., 2013). In the light of the recent report suggesting that Rus and AcoP are copper resistance determinants in *A. ferrooxidans* ATCC 23270 (Navarro *et al*., 2016), it will be of interest to study whether these proteins have a similar role in copper resistance in *A. ferrivorans*.

González *et al*. (2014) reported the presence of a *ppk2* in a GI of *A. ferrivorans* SS3. The protein comparisons shown in Fig. 1 support the presence of this PPK2 (with 89% Blastp score) and also indicate that *A. ferrivorans* SS3 contains a PPX protein (85% Blastp score), suggesting that the polyP‐dependent copper resistance system may also be operative in this psychrotolerant bacterium.

It is also important to consider that not only a higher presence of genes related to metal resistance is the only factor important for extreme metal tolerance but also metal speciation and pH are also important to consider (González *et al*., 2014). In addition, differences in cell envelope components also may influence metal resistance in bacteria (Harrison *et al*., 2007; Almárcegui *et al*., 2014a).

### Acidithiobacillus thiooxidans

Studies on copper tolerance mechanisms in *A. thiooxidans* have also been approached recently (Wen *et al*., 2014). A putative MCO gene (*cueO*) was annotated in the genome of *A. thiooxidans* ATCC 19377 (Valdes *et al*., 2011). This gene is also present in *Acidithiobacillus caldus* ATCC 51756, *Thiomonas arsenitoxydans* and less likely in *Alicyclobacillus acidocaldarius* LAA1 but is absent in *A. ferrooxidans* and all the other microorganisms listed in Fig. 1. The MCO has been studied in detail in *E. coli* (Grass and Rensing, 2001; Roberts *et al*., 2002; Hoegger *et al*., 2006). The transcriptional level of *A. thiooxidans cueO* was upregulated in response to 10 mM copper sulfate and a response to copper of a putative *cueO* promoter was detected, suggesting it might be stimulated by a putative CueR protein (Wen *et al*., 2014). By using a modified markerless gene disruption system, these authors generated a *cueO* mutant of *A. thiooxidans*. This mutant was more sensitive to external copper and this activity could be restored after complementing the *cueO* gene, strongly suggesting the involvement of *cueO* in copper tolerance of this bacterium (Wen *et al*., 2014).

Figure 1 shows that *A. thiooxidans* contains several genes coding for proteins with a high identity Blastp score (>70%) with those of the CusCBA systems from *A. ferrooxidans*. Furthermore, *A. thiooxidans* may have two Cus systems with similar genetic contexts and the characteristic domains of the previously characterized Cus systems (Fig. 3).

*Acidithiobacillus thiooxidans* also showed a protein with a high Blastp score (>65%) with CopB from *A. ferrooxidans* (Fig. 1). This putative copper ATPase shares several domains with CopB_Af_ such as two heavy metal‐binding domains (HMA) and an ATPase E1‐E2 domain, which could be related with the incoming and exporting of copper.

Moreover, Fig. 1 indicates that *A. thiooxidans* genome has a gene coding for a protein similar to the copper chaperone CopZ from *B. subtilis*. The *A. thiooxidans* protein contains the same heavy metal‐binding domain present in CopZ_*B. subtilis*_ with conserved copper‐binding amino acids (MXCXXC). In addition, the bioinformatics analysis of its subcellular location predicted it to be a putative cytoplasmic copper chaperone, suggesting it could be an *A. thiooxidans* CopZ. Upstream of the gene coding for this protein, there is a putative heavy metals transcriptional regulator that could regulate the expression of this possible *A. thiooxidans* cytoplasmic metal chaperone (Fig. 3).

As Fig. 1 shows, *A. thiooxidans* has a high Blastp (>78%) with the genes coding for PPK and PPX from *A. ferrooxidans* and they share basically the same structural domains. Considering that *A. thiooxidans* accumulates polyP granules as seen by TEM (Orell *et al*., 2010), it is possible that a functional polyP‐dependant metal resistance system similar to that previously proposed for *A. ferrooxidans* (Alvarez and Jerez, 2004) is also present in *A. thiooxidans*.

The genome sequence of an *A. thiooxidans* Licanantay strain isolated from a biomining operation suggested the presence of GIs (Travisany *et al*., 2014) as it has been previously reported for *A. ferrooxidans* ATCC 53993 strain (Orellana and Jerez, 2011) and for *A. ferrivorans* SS3 with a predicted GI in its genome (González *et al*., 2014). Most likely, horizontal gene transfer in mining environments probably generates these genomic elements.

### Acidithiobacillus caldus

Regarding copper tolerance of *A. caldus*, a copper MIC of 24 mM has been reported for strain DSM8584 (Watkin *et al*., 2008). The response to copper stress of an *A. caldus* strain isolated in China has been analysed (Xia *et al*., 2010). The authors found a negative effect on the activity of some of the enzymes involved in sulfur metabolism, such as sulfite oxidase and APS reductase. Nevertheless, no direct evidence was reported to support the idea of overexpression of a Cu‐specific inducible ATPase pump; the authors speculated that would be present under their conditions of study.

*Acidithiobacillus caldus* ATCC 51756 (Mangold *et al*., 2013) (see Fig. 1) and strain SM‐1 (You *et al*., 2011) contain a variety of genes coding for putative copper resistance‐related proteins as they showed a great degree of identity (between 50% and 90%) with those known for *A. ferrooxidans* (Navarro *et al*., 2009; Orellana and Jerez, 2011). These two strains of *A. caldus* share several of these genes in their chromosomal DNA, such as the components of an RND‐type efflux pump (CusABC), two P‐type ATPases (CopB), two cytoplasmic metalochaperones (CopZ) and a copper oxidase (CueO) (see Fig. 1). Interestingly, strain SM‐1 contains a gene coding for a putative periplasmic copper chaperone (CusF), which has been described in *E. coli* (Loftin *et al*., 2005) and in *A. ferrooxidans* ATCC strains 23270 and 53993 (Orell *et al*., 2010; Orellana and Jerez, 2011).

The search in *A. caldus* of possible copper resistance determinants similar to those of the CusCBAF system from *A. ferrooxidans* showed possible homologue proteins although in general they had 40–60% Blastp score (Fig. 1). These proteins possess the characteristic structural domains of these efflux systems and their corresponding genes are contiguous in the genome (Fig. 3). Most likely, they could be related to the transport of heavy metals such as copper, zinc or cadmium.

In addition, *A. caldus* showed two ATPases with copper‐binding sites (Interpro IPR006122) and with a high Blastp score (>50%) when compared with CopB1 from *A. ferrooxidans*. Interestingly, a possible cytoplasmic copper chaperone with high similarity to CopZ_*B. subtilis*_ was present in the same DNA region, close the two ATPases already mentioned (Fig. 3). Figure 3 also shows a glutaredoxin in the context of one of the ATPases that could be related to maintain the redox state of the sulfhydryl groups present in cellular proteins damaged by the presence of copper (Almárcegui *et al*., 2014b).

Figure 1 shows the possible presence of a MCO in *A. caldus*. This putative protein contains the same domains described for *E. coli* CueO as noted before (Navarro *et al*., 2013).

Although a small score (5–10%) indicated the possible presence of Rus and AcoP in *A. caldus* (Fig. 1), a detailed analysis of the amino acid sequences of these two putative periplasmic proteins indicated that none of them have the domains and copper‐binding sites characteristic of the respirasome‐related proteins from *A. ferrooxidans*.

As seen in Fig. 1, A*. caldus* shows the likely presence of PPX and PPK similar to those from *A. ferrooxidans* (Blastp score >70%), both involved in polyP metabolism and the polyP‐related metal resistance system reported before for *A. ferrooxidans* (Alvarez and Jerez, 2004; Orell *et al*., 2010). It has been reported that *A. caldus* accumulates polyP granules as many other acidophilic microorganisms with high metal resistance (Orell *et al*., 2012). Furthermore, both *A. ferrooxidans* and *A. caldus* have a putative Pho regulon, which includes *ppx* in its genomic context (Vera *et al*., 2003; Mangold *et al*., 2013; Navarro *et al*., 2013). It is possible then that both acidophilic bacteria would respond in a similar way to the presence of copper (Navarro *et al*., 2013).

### Leptospirillum ferriphilum

*Leptospirillum ferriphilum* ML‐04 has some identity (20 to 50% depending on the Cus protein) with a CusCBAF system from *A. ferrooxidans* (Fig. 1) and two copies of this system are present in its genome. As seen in Fig. 3, both Cus systems have a genetic context similar to that described for *E. coli*, except that CusF is neighbour of CusA. Nevertheless, all of the components of these putative Cus systems contain the characteristic domains of these metal efflux pumps. On the other hand, a copper ATPase with some identity to CopA_Af_ and a gene coding for a protein with 30% identity to CopZ_*B. subtilis*_ were also found. However, this last protein would rather correspond to a mercury reductase and it contains a metal‐binding domain similar to that present in CopZ. Finally a putative PPX coding gene was found in *L. ferriphilum* but in a different genomic context. In addition, a putative PPK2 was also found in this bacterium but with a low score when compared with the equivalent enzyme from *A. ferrooxidans*. Without current information of the existence of polyP in *L. ferriphilum* and the low identity score found for both possible enzymes involved in polyP metabolism, at present it is not possible to speculate on their putative role in metal resistance in this bacterium.

### Leptospirillum ferrooxidans

The iron oxidizer *L. ferrooxidans* shows several putative copper resistance determinants similar to those in *A. ferrooxidans* (Fig. 1). However, its iron‐oxidizing system lacks rusticyanin (Blake *et al*., 1993). As seen in Fig. 1, the iron oxidizer *L. ferrooxidans* contains a possible CusCBAF in which its CusA has a Blastp score over 50% when compared with CusA1 from *A. ferrooxidans*. In addition, the genetic context of the *L. ferrooxidans* is in general similar to those described for these Cus systems in other bacteria (Fig. 3). Compared with *L. ferriphilum*,* L. ferrooxidans* has only one Cus system. In addition, *L. ferrooxidans* genome contains a possible metal ATPase with a Blastp score higher than 20% when compared with *A. ferrooxidans* CopA. Although this is not a very high score value, this protein conserves the metal binding (HMA), E1‐E2_ATPase and ZntA domains typical of CopA.

Regarding the possible existence of a polyP‐dependent metal resistance system in *L. ferrooxidans*, the bacterium showed two proteins with a low Blastp score (over 10%) compared with the *A. ferrooxidans* PPX. These two possible PPX enzymes have the Ppx/GppA (IPR003695) domain characteristic of these proteins. On the contrary, no PPK similar proteins were found. This evidence and the lack of information for the presence of polyP do not allow us to suggest the existence of a metal resistance system dependent on polyP in this microorganism.

### Acidimicrobium ferrooxidans

The bioinformatics analysis in Fig. 1 indicates that *A. ferrooxidans DSM 10331* has a possible copper ATPase with a BlastP score higher than 20% when compared with CopA1 from *A. ferrooxidans*. Both proteins share several important domains, such as the metal‐binding domain. Interestingly, next to the gene for this putative ATPase the gene coding for a putative small cytoplasmic chaperone similar to CopZ with a copper‐binding site (MXCXXC) characteristic of this protein was found by using the CELLO v.2.5 program (Fig. 3). Being a Gram positive, this bacterium does not contain genes with identity to the Cus system of *A. ferrooxidans* in Fig. 1.

*Acidimicrobium ferrooxidans* also has genes coding for putative PPX and PPK both possessing the characteristic domains of these enzymes. Although the presence of inorganic polyP has not been reported in this bacterium, most likely it exists as it occurs in other biomining bacteria to play an important role for oxidative stress response and metal resistance (Orell *et al*., 2010; Navarro *et al*., 2013).

### Sulfobacillus thermosulfidooxidans

This Gram (+) bacterium is not expected to have a complete Cus system. Figure 1 shows the gene for one protein with a low identity to CusA1 from *A. ferrooxidans* and no other possible components of a Cus system. However, this possible protein does not contain any of the domains present in CusA1. In contrast, *S. thermosulfidooxidans* showed four possible ATPases with similarity to CopA1 and CopB1 proteins from *A. ferrooxidans*. All these putative copper ATPases possess metal binding (HMA), E1‐E2_ATPase and ZntA domains, all of them important for the function of the ATPases and which also form part of CopA_Af_ and CopB_Af_. As seen in other bacteria (Radford *et al*., 2003; Navarro *et al*., 2016), one of these ATPAses is found contiguous to a putative CopZ having an MXCXXC copper‐binding site characteristic of these cytoplasmic chaperones (Fig. 3).

As seen in Fig. 1, *S. thermosulfidooxidans* has a gene coding for a protein with 20–30% Blastp score compared with AcoP from *A. ferrooxidans*. There are three copies of this gene coding for a protein that contains a cupredoxin domain to bind copper. However, although these cupredoxin protein genes are neighbours of cytochromes, no *rus* gene is present in this Gram (+) iron oxidizer (Blake *et al*., 1993).

Finally, as seen in Fig. 1, clearly Gram (+) bacteria do not contain the proteins related to the Cus systems due to their different envelope structure compared with the Gram (−) bacteria. On the other hand, most Gram (+) bacteria, as expected do not show the presence of periplasmic Rus.

## Copper tolerance of biomining Archaea

Concerning archaeal copper resistance mechanisms, a few metal efflux pumps have been identified from sequenced genomes of some members of this domain (Pedone *et al*., 2004; Wheaton *et al*., 2015; see Fig. 1). A Cu resistance (*cop*) loci has been described in *Archaea*, which includes genes encoding for a new type of archaeal transcriptional regulator (*CopT*), a putative metal‐binding chaperone (*CopM*) and a putative Cu‐transporting P‐type ATPase (*CopA*) (Ettema *et al*., 2006) (see Fig. 2B).

Single unit, membrane class 1B heavy metal translocating P‐type ATPases are involved in homeostasis and resistance to copper as well as to other metals (Palmgren and Nissen, 2011). Although *Sulfolobus solfataricus* is not a biomining microorganism, it has been considered a model for archaeal studies. The copTMA operon from *S. solfataricus* P2 (i.e., copRTA in *S. solfataricus* 98/2 as reported by Villafane *et al*., 2009) contains a copper‐responsive regulator (CopT), a copper‐binding protein (CopM) that includes the metal coordinating ligands within the so‐called TRASH domain (‘Trafficking, Resistance and Sensing of Heavy metals’) (Ettema *et al*., 2003).

CopT usually has an HTH (‘helix–turn–helix’) DNA binding domain in its N‐ terminal end and a TRASH metal‐binding domains in their C‐terminal ends. On the other hand, both CopA and CopM may have metal‐binding domains such as TRASH, YHS (tyrosine, histidine, serine) or HMA (heavy metal‐associated) (Gitschier *et al*., 1998).

CopA and CopT copper resistance systems are present in most of the selected archaea as shown in Fig. 1. In several cases, there was an overlap between CopA and CopM at the genomic level and therefore they are not annotated in the protein databases. For this reason, only CopA from *S. solfataricus* was included for comparative purposes in Fig. 1.

CopA is known to impart copper resistance to *S. solfataricus* (Ettema *et al*., 2006), being an effective copper pump at low copper concentrations. Other archaeal acidophiles also contain versions of the *cop* operon (Fig. 1). The search for possible CopT transcriptional regulators in the genomes of the archaea included in Fig. 1 was done by comparison with CopT from *S. solfataricus*. All archaea where CopT was identified showed high amino acid sequence identity compared with the *S. solfataricus* regulator and all of these putative transcriptional regulators contained the TRASH and HTH domains characteristic of CopT_*S. solfataricus*_.

The genome of *Ferroplasma acidarmanus* contains a cop operon (copYZB) with similar structure and response to copper as for copTMA (Baker‐Austin *et al*., 2005). All the putative ATPases identified in the genomes of *F. acidarmanus*,* T. acidophilum*,* T. volcanium*,* P. torridus* and *A. cupricumulans* in Fig. 1 contain TRASH domains (data not shown). On the other hand, all the possible CopA found in archaeal genomes in Fig. 1 contained YHS or HMA metal‐binding domains (data not shown).

*In silico* studies have further identified a CPx‐ATPase, which most likely mediates the efflux of heavy metal cations in the biomining archaeon *M. sedula* (Auernik *et al*., 2008). This putative protein has significant identity to a P‐type ATPase from *S. solfataricus* (CopA) (Ettema *et al*., 2006). Moreover, *M. sedula* contains ORFs with significant similarity to both *CopM* (Msed0491) and *CopT* (Msed0492) from *S. solfataricus* (Auernik *et al*., 2008). Maezato *et al*. (2012) reported a genetic approach to investigate the specific relationship between metal resistance and lithoautotrophy during biotransformation of CuFeS_2_ by *M. sedula*. The functional role of its *copTMA* operon was demonstrated by cross‐species complementation of a Cu‐sensitive *S. solfataricus copR* mutant (Maezato *et al*., 2012).

The previously published genetic context of several archaeal putative CopMA and CopT showed that all of them have the same organization (Ettema *et al*., 2006; Maezato *et al*., 2012; Orell *et al*., 2013). Some of the archaea analysed in Fig. 1 also possess the same genetic organization, indicating that this copper resistance system is highly conserved in most archaea and it could be one of the most relevant ones.

Regarding the polyP‐based copper resistance mechanism, *S. metallicus* is known to tolerate very high concentrations of copper and accumulates high amounts of polyP granules. Furthermore, the levels of intracellular polyP are greatly decreased when this archaeon is either grown in 200 mM Cu or shifted to 100 mM Cu and an increase in exopolyphosphatase (PPX) activity and Pi efflux due to the presence of Cu suggests a metal tolerance mechanism mediated through polyP in this archaeon (Remonsellez *et al*., 2006; Orell *et al*., 2010, 2012).

*Acidithiobacillus ferrooxidans* (Alvarez and Jerez, 2004) and *S. metallicus* (Remonsellez *et al*., 2006) apparently do not contain PitA in their genomes but they have Pho84‐like phosphate transporters similar to Pho‐84 from *Saccharomyces cerevisiae* which transports metal‐phosphate complexes at acid pH (Fristedt *et al*., 1999) (see Fig. 1).

Figure 1 clearly shows that all Chrenarchaoeta analysed had a high Blastp score for their putative PPX when compared with the PPX from *S. solfataricus*. All of these putative PPX enzymes contain the characteristic domains of this PPX (data not shown). Interestingly, all Euryarchaeota in Fig. 1 do not show a protein with identity to PPX from *S. solfataricus*. This is due to the fact that Euryarchaeota apparently do not have PPX genes identified in their genomes and Crenarchaeota have PPX genes but no genes coding for PPK. As they do synthesize polyP (Orell *et al*. (2012), a different unknown PPK yet to be identified should be present in these archaeons.

It was recently reported that the wild‐type *M. sedula* (DSM 5348T) is a *pitA* mutant, as *M. sedula* strain CuR1, a spontaneous mutant, had a supranormal metal resistance and was able to leach copper from CuFeS_2_ at an accelerated rate (McCarthy *et al*., 2014). This mutant contained a gene orthologue of the bacterial *pitA. M. sedula* PitA was a low affinity, high velocity secondary transporter implicated in copper resistance and arsenate sensitivity. This archaeal PitA protein would then be a key element for the increased metal resistance of CuR1 and its increased capacity to leach copper (McCarthy *et al*., 2014). This archaeal Pit transport system would support increased phosphate efflux and metal symport through this Pit‐like system (see Fig. 2B). Previous studies on metal resistance in *S. metallicus* proposed that another phosphate transporter (Pho84 like) would be present (Remonsellez *et al*., 2006). Like Pit, Pho84 belongs to the family of phosphate‐proton symporters and the major facilitator superfamily (MFS). As suggested by McCarthy *et al*. (2014) perhaps PitA and Pho84 comprise dual phosphate transporters that are inherent to these thermoacidophilic archaeons and are critical for their extreme metal resistance.

All archaeons analysed had proteins with around 20% identity to Pho84. Most of these putative proteins are annotated as major facilitator superfamily (MFS) transporters as they contain an MFS domain. In addition, all of them contain a domain annotated as ‘phosphate:H+ symporter’. Considering that both domains are present in Pho84 and that there is evidence for the presence of Pho84‐like and PitA orthologues (Fig. 1) in some archaeons (Remonsellez *et al*., 2006; McCarthy *et al*., 2014), the possible function of polyP in metal resistance in these analysed microorganisms cannot be discarded.

Actual evidence suggests that polyP may provide mechanistic alternatives in tuning microbial fitness for the adaptation under stressful environmental situations such as oxidative stress may be of crucial relevance among extremophiles. This is supported by the recent report assigning the universally conserved biopolymer general roles as a protein‐protective chaperone and in regulating general stress response pathways (Gray *et al*., 2014; Gray and Jakob, 2015).

Figure 1 shows that proteins from some archaeal microorganisms (*M. sedula*,* T. acidophilum*,* T. volcanium*) had a BlastP score of around 20% when compared with the blue‐copper protein Rus from *A. ferrooxidans*. Other microorganisms such as *S. metallicus* showed a lower identity (10%). All these archaeal proteins have been annotated as possible rusticyanin, having a type I copper‐binding site and a characteristic cupredoxin domain. In *M. sedula*, one of these proteins has been suggested to be involved in iron oxidation (Auernik *et al*., 2008). The proteins from *M. sedula*,* S. acidocaldarius*,* S. tokodai* and *S. solfataricus* having identity with *A. ferrooxidans* Rus are sulfocyanins which also have a copper‐binding site. The genetic context for the genes coding these proteins is not identical to that of *rus* gene from *A. ferrooxidans* but are close to cytochromes and iron‐sulfur protein genes. As mentioned before, some experimental evidence supports the role of Rus as a possible copper chaperone related with copper resistance in *A. ferrooxidans* (Navarro *et al*., 2016). However, whether some of the putative Rus proteins from Fig. 1 other than Rus from *A. ferrooxidans* are implicated in copper resistance in other bacteria and archaea clearly remains to be demonstrated.

Finally, proteomic studies of *S. metallicus* subjected to copper, showed 23 downregulated and 30 upregulated proteins, suggesting they are possibly involved in copper resistance (Orell *et al*., 2013). A similar behaviour was also observed in ‘*Fp. acidarmanus*’ challenged by either arsenite or copper (Baker‐Austin *et al*., 2005; Baker‐Austin and Dopson, 2007). Most of these proteins are related to the production and conversion of energy, amino acids biosynthesis and stress responses. Within proteins upregulated in *S. metallicus* cells exposed to copper, a putative ATP synthase subunit B was detected (Orell *et al*., 2013). When cells are subjected to some stressing conditions such as the presence of heavy metals, a greater cellular demand for energy has been reported to occur (Baker‐Austin *et al*., 2005).

Several previous reports have suggested that oxidoreductases contribute to an oxidative protection in both bacteria and archaea in response to heavy metals (e.g. Rodriguez‐Montelongo *et al*., 2006; Williams *et al*., 2007). Some proteins involved in oxidative damage repair, such as NADH‐dependent oxidases and thioredoxin reductases, were expressed in cells of ‘*Fp. acidarmanus*’ exposed to arsenite (Baker‐Austin and Dopson, 2007). The expression of this group of proteins has also been observed when the same microorganism was exposed to copper (Baker‐Austin *et al*., 2005).

## Concluding remarks

Current knowledge indicates that in addition to passive adaptations such as a positive membrane potential and a very high number of basic proteins in the periplasm of Gram (−) bacteria that make difficult cation displacements, key active elements involved in metal resistance in environmental acidophilic microorganisms appear to be a wide repertoire of known regulated copper resistance determinants similar to those present in all microorganisms. The duplications of these copper resistance genes also confer extra metal tolerance to the microorganisms containing them. Global OMICS procedures have allowed determining the presence of novel copper‐chaperones and other possible metal resistance determinants by their overexpression in the presence of the tested metal. The study of novel metal resistance determinants in environmental microorganisms will also help with the functional annotation of these genes in the increasingly available genomic sequences from environmental extremophilic bacteria and archaea.

Horizontal gene transfer by means of mobile genetic elements such as GIs plays an important role in increasing the adaptability and versatility of microorganisms living in environments with high metal concentrations. Most importantly, is to consider that studies of metals resistance in individual microorganisms do not necessarily reflect some interspecific bacterial cooperation that takes place during metal resistance in complex communities. Therefore, future studies on metal resistance determinants in biomining microorganisms should also include detailed analysis of the consortia actually living in industrial bioleaching operations to thoroughly understand the mechanisms that acidophilic bioleaching microorganisms use to adapt to their extreme environments. This knowledge should eventually improve biomining or metal bioremediation processes if the most fitted bacteria are involved in these industrial processes.

## Conflict of interest

None declared.
